# Potassium-competitive acid blockers for *Helicobacter pylori* eradication in pediatrics: a narrative review of pharmacologic rationale, clinical evidence, and practical considerations

**DOI:** 10.3389/fcimb.2026.1810850

**Published:** 2026-05-13

**Authors:** Bingyan Gu, Jingying Le, Haifang Cai, Liwei Liu

**Affiliations:** 1 The First School of Clinical Medicine, Zhejiang Chinese Medical University, Hangzhou, China; 2 The Second Clinical Medical College of Zhejiang Chinese Medical University, Hangzhou, China; 3 The First Affiliated Hospital of Zhejiang Chinese Medical University (Zhejiang Provincial Hospital of Chinese Medicine), Hangzhou, China

**Keywords:** eradication, Helicobacter pylori, pediatrics, potassium-competitive acid blockers, vonoprazan

## Abstract

*Helicobacter pylori* (*H. pylori*) infection remains highly prevalent worldwide and is strongly associated with chronic gastritis, peptic ulcer disease, and an elevated risk of gastric cancer. In pediatric clinical practice, eradication therapy is challenged by rising antibiotic resistance, inadequate acid suppression, and poor adherence to complex multidrug regimens. Potassium-competitive acid blockers (P-CABs) represent a newer class of antisecretory agents that competitively and reversibly inhibit gastric H^+^/K^+^-ATPase, resulting in a more rapid onset and more sustained control of intragastric pH compared with conventional proton pump inhibitors (PPIs). P-CABs offer greater flexibility in dosing timing relative to meals in children, with substantial potential to simplify treatment regimens and improve *H. pylori* eradication rates in the pediatric population. This narrative review summarizes the pharmacologic rationale for using P-CABs in pediatric *H. pylori* eradication, synthesizes current clinical evidence—derived mainly from Japanese observational studies of vonoprazan (VPZ)—and discusses key issues regarding resistance, tolerability, and practical clinical application. Published pediatric studies, predominantly involving adolescents and older pediatric cohorts, suggest that VPZ-based regimens may achieve favorable eradication rates, while short-term tolerability appears broadly similar to that of PPI-based therapies in the available comparative data. However, the existing evidence is limited by small sample sizes, narrow geographic scope, and marked heterogeneity in study design, antibiotic backbones, and resistance patterns, which preclude direct cross-study comparisons. Data supporting other P-CABs in pediatric populations remain very scarce. Future research should prioritize pediatric pharmacokinetic/pharmacodynamic studies to optimize weight- and age-based dosing, conduct resistance-stratified comparative trials, and perform cost-effectiveness analyses. Until more robust pediatric evidence becomes available, P-CAB-based regimens may be considered a viable option in selected clinical scenarios, while clinicians should exercise caution when extrapolating adult data and remain vigilant toward local antibiotic resistance epidemiology.

## 1 Introduction

*Helicobacter pylori* (*H. pylori*) infection is typically acquired in childhood and remains prevalent worldwide. The global overall prevalence rate of *H. pylori* infection in children is 35.1%, while in China, it ranges from 2.9% to 46.3% (Wang et al., 2024; Xu et al., 2024). Infection in children is not only closely associated with gastrointestinal disorders such as chronic gastritis and peptic ulcers but may also impair nutrient absorption, thereby interfering with normal growth and development (Zheng et al., 2025; Krauthammer et al., 2026). Meanwhile, it may also lead to significant gastric mucosal precancerous lesions in pediatric patients, such as atrophic gastritis and intestinal metaplasia (M et al., 2022; Abdun et al., 2025). Although the incidence of gastric cancer (GC) in children is low, cases of *H. pylori*-associated GC have been reported (Li et al., 2025). Eradicating *H. pylori* can prevent gastrointestinal complications, reduce bacterial transmission, and alleviate parental anxiety (Homan et al., 2024; Aguilera-Matos et al., 2025). However, in children, treatment decisions should be guided by evidence-based indications rather than infection status alone. According to the Updated Joint ESPGHAN/NASPGHAN Guidelines (2023) (Homan et al., 2024), eradication therapy is mandatory for children with *H. pylori* infection complicated by peptic ulcer, gastric MALT lymphoma, or GC. It is also recommended, following individualized assessment, for cases of refractory iron deficiency anemia (after excluding other causes), individuals with a family history of GC in first-degree relatives, and those with chronic active gastritis accompanied by significant symptoms who are *H. pylori*-positive (Homan et al., 2024; Le Thi et al., 2024).

Standard pediatric regimens have historically relied on proton pump inhibitor (PPI)-based triple therapy (Malfertheiner et al., 2007; Homan et al., 2024). However, this approach faces significant challenges, including high rates of antimicrobial resistance and suboptimal eradication rates with empirical treatment (Wen et al., 2017). PPIs work by inhibiting gastric acid secretion and elevating intragastric pH, thereby creating a suitable environment for antibacterial agents to exert their effects. Nevertheless, traditional PPIs have notable limitations. PPIs require activation in acidic canaliculi and are sensitive to dosing timing and CYP2C19-mediated metabolism, which can lead to variable acid suppression (Andersson and Carlsson, 2005). Moreover, missed doses or incorrect timing further reduce efficacy in children.

Potassium-competitive acid blockers (P-CABs) inhibit the gastric H+/K+-ATPase via competitive, reversible potassium blockade, producing faster onset and more sustained acid suppression than PPIs (Andersson and Carlsson, 2005). In recent years, P-CABs have demonstrated favorable efficacy and safety in treating *H. pylori* infection in adults (Rokkas et al., 2025). The updated AGA Clinical Practice Update highlights P-CABs as an important option in adult eradication regimens (Patel et al., 2024), offering a new perspective for treating pediatric *H. pylori* infection. Although there is growing interest in the efficacy and safety of P-CABs for eradication therapy in children, comprehensive review studies on this topic are still scarce.

This focused narrative review aims to (i) clarify the pharmacologic rationale for P-CAB-based eradication in children, (ii) summarize currently available pediatric clinical evidence, primarily concerning vonoprazan (VPZ), and (iii) provide a reference for optimizing anti-*H. pylori* treatment regimens, improving eradication rates, and identifying directions for future research. Throughout the manuscript, adult data are cited only when pediatric evidence is unavailable and are explicitly identified as such.

## 2 Methods

This manuscript is a focused narrative review informed by a targeted literature search rather than a systematic review or meta-analysis. We searched PubMed, Embase, and Web of Science for studies published within the past 10 years using combinations of “*Helicobacter pylori*”, “pediatrics” or “adolescents” or “children” with “potassium-competitive acid blockers” or “Vonoprazan” or “Tegoprazan” or “Keverprazan” and also consulted relevant international guidelines and consensus statements. Priority was given to pediatric clinical studies when discussing efficacy, safety, and practical treatment considerations. Adult studies were cited only to support pharmacologic rationale or to provide indirect contextual evidence when pediatric data were unavailable, and were explicitly identified as adult evidence. Given the limited and heterogeneous pediatric literature, no formal pooled statistical analysis or structured risk-of-bias assessment was performed.

## 3 Clinical challenges of *H. pylori* eradication in pediatrics

The treatment of *H. pylori* infection in children presents significant distinctiveness and clinical challenges. Compared with adults, children exhibit a significantly lower eradication rate and a higher recurrence rate (Xu et al., 2024; Homan et al., 2025), which can be attributed to multiple influencing factors (Riaz et al., 2025; Tibasima et al., 2025).

Firstly, the digestive system in children is not fully mature, and their hepatic metabolic and renal excretory functions are relatively weaker, resulting in substantially lower drug tolerance than in adults. Expert consensus guidelines indicate a limited selection of antibiotics suitable for use in children (Homan et al., 2024). Tetracyclines are contraindicated in children under 8 years old due to their adverse effects on tooth development, and fluoroquinolones are generally not recommended for children owing to potential cartilage toxicity. Consequently, the primary available options are amoxicillin (AMO), clarithromycin (CLA), and metronidazole (MET), which complicates the design of effective treatment regimens.

Secondly, antibiotics exhibit high and regionally variable resistance. In some countries, the resistance rate to CLA in children can even exceed that observed in adults (Agudo et al., 2010), a phenomenon potentially linked to the more frequent use of this antibiotic class in children for conditions like respiratory infections (Agudo et al., 2009). A global systematic review encompassing data from 2000 to 2023 revealed primary resistance rates in children as follows: MET 35.3%, CLA 32.6%, levofloxacin 13.2%, AMO 4.8%, and tetracycline 2.1%, which also noted a trend of increasing antibiotic resistance across most WHO regions (Salahi-Niri et al., 2024). In southeast China, resistance rates among children were reported as 32.8% for CLA, 81.7% for MET, and 22.8% for levofloxacin, while resistance to AMO remained lower (Shu et al., 2022). High rates of antimicrobial resistance compromise the efficacy of empiric eradication regimens. A meta-analysis by Wen et al. showed an eradication rate of only 71% (647/911) for empirical triple therapy containing CLA or MET in Asian pediatric patients (Wen et al., 2017). Although susceptibility testing is recommended when feasible, obtaining cultures or molecular resistance data usually requires endoscopy, which is not universally performed in children.

Thirdly, standard regimens typically involve 3–4 medications, which may have unpleasant tastes. Significant gastrointestinal side effects, such as the bitter taste and nausea associated with MET, can adversely affect treatment adherence (Sa et al., 2025). Irregular medication intake not only predisposes to treatment failure but may also contribute to the development of resistant *H. pylori* strains, thereby increasing the difficulty of subsequent therapies. Some medication tablets are too large for children to swallow easily. In contrast, P-CABs like VPZ are often formulated in smaller tablets, which are more suitable for pediatric administration (Cardenas Fernandez et al., 2025).

Given these constraints, an ideal pediatric eradication regimen should not only involve antibiotic selection based on drug susceptibility testing but also pursue a simplified administration schedule to improve medication adherence, while providing potent and stable acid suppression to optimize the bactericidal environment for antibiotics. P-CABs exhibit unique potential in these aspects and are expected to serve as a novel strategy for optimizing eradication therapy in children.

## 4 Pharmacologic rationale and overview of P-CABs

### 4.1 P-CABs relevant to *H. pylori* treatment

Since the introduction of P-CABs in Japan in 2015 (Jenkins et al., 2015), several P-CABs have been launched or entered clinical application worldwide. Among them, only VPZ (Song et al., 2025), Tegoprazan (Park et al., 2026), and Keverprazan (Tan et al., 2024) are currently utilized for *H. pylori* eradication. Although P-CABs have demonstrated excellent efficacy in treating *H. pylori* infection in adults and are approved as components of adult eradication regimens in some countries (Shirley, 2024), their application in pediatric populations remains in the exploratory phase. Presently, no P-CAB has received formal regulatory approval for the specific indication of *H. pylori* eradication in children. Furthermore, the majority of clinical research involving children has been conducted in Japan, and nearly all of these studies have focused on VPZ (Kakiuchi et al., 2019; Kakiuchi et al., 2023). This may be attributed to the fact that Japan, as a country with a high incidence of GC, has adopted a more proactive eradication strategy, and its guidelines recommend eradicating asymptomatic *H. pylori*-positive adolescents to prevent future GC (Isomoto et al., 2026). In contrast, clinical evidence from other regions is very limited. However, overall, the use of P-CABs in pediatrics is mostly off-label, requiring more clinical evidence for support.

### 4.2 Pharmacological characteristics compared with PPIs

Although both P-CABs and PPIs target the parietal cell H^+^/K^+^-ATPase, their mechanisms of action are fundamentally distinct, leading to differentiated clinical value in the treatment of *H. pylori* infection in children. Compared with PPIs, P-CABs achieve near-maximal acid suppression from the first dose, sustain higher intragastric pH over 24 hours, and can be administered without strict pre-meal timing (Visaggi and Savarino; Shang et al., 2025). These pharmacologic properties translate into reduced dosing complexity and may therefore better accommodate the practical challenges of pediatric medication administration. Furthermore, P-CABs have a longer half-life of 4–9 hours (Visaggi and Savarino). Their reversible binding allows for repeated interaction with the pump while plasma concentrations are sufficient, enabling stable and sustained 24-hour acid suppression. Studies have shown that VPZ 20 mg administered twice daily for 7 days can achieve intragastric pH ≥ 4 and ≥ 5 for 100% and 99% of the time, respectively (Kagami et al., 2016), creating a favorable low-acid environment for *H. pylori* eradication. Since consistent elevation of gastric pH enhances the antimicrobial activity of co-administered antibiotics, this more potent and stable pH control may improve eradication rates, particularly in scenarios of suboptimal adherence.

Adequate acid suppression is a key determinant of eradication success (Malfertheiner et al., 2007). Higher intragastric pH improves the stability and activity of several antibiotics used in eradication therapy and may increase the proportion of bacteria in a metabolically active state that is more susceptible to antibiotics (Shang et al., 2025). Conversely, in a strongly acidic environment, bacterial metabolism slows, and the bacteria enter a latent or dormant state, reducing their sensitivity to antibiotics. Gastric emptying time and intestinal transit speed are generally faster in children than in adults, which may affect the residence time and absorption of orally administered antibiotics in the digestive tract. An overly short gastrointestinal transit time can shorten the duration of effective drug concentration maintenance. Consequently, some scholars suggest that in pediatric eradication therapy, maintaining a higher intragastric pH is important not only for bacterial sensitivity but also for potentially prolonging the presence of antibiotics such as AMO and CLA in the gastrointestinal tract (Kakiuchi, 2023). The stronger acid suppression by P-CABs and their potential to reduce antibiotic-associated diarrhea may contribute to achieving this goal (Shang et al., 2025).

Inter-individual variability in PPIs exposure is partly driven by CYP2C19 genotype, which can translate into variable acid suppression (Ma et al., 2025). P-CABs are less dependent on CYP2C19 metabolism, which may reduce variability in acid control relative to PPIs (Visaggi and Savarino). During childhood, hepatic enzyme activity varies with age, with CYP2C19 and CYP3A4 generally more active than in adults (Kakiuchi, 2023). This suggests that children may metabolize and clear PPIs more rapidly. Fortunately, P-CABs are less dependent on CYP2C19. Therefore, even with potentially faster drug metabolism in children, their plasma concentrations and efficacy may be less affected by genetic variation than those of conventional PPIs.

However, whether this pharmacologic advantage meaningfully improves pediatric eradication outcomes requires direct pediatric comparative studies.

## 5 Clinical evidence of P-CAB-based eradication regimens in pediatrics

Most available pediatric clinical evidence centers on VPZ, with marked heterogeneity across studies. Therefore, when interpreting eradication rates, it is essential to account for variations in antibiotic regimens, treatment duration, local resistance rates, outcome definitions (intent-to-treat [ITT] vs. per-protocol [PP]), and the timing of follow-up testing.

**Figure 1:**
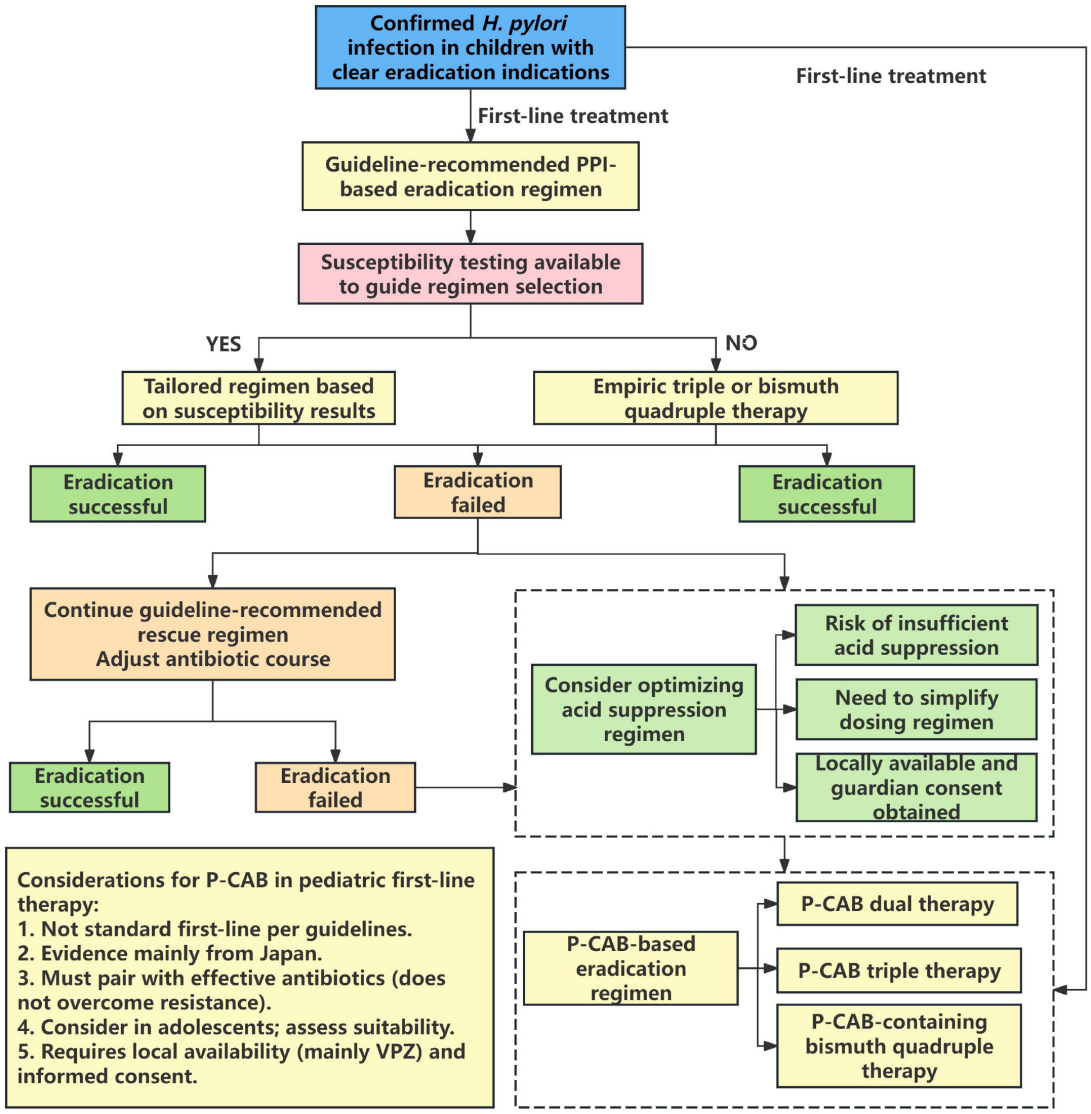
Clinical decision pathway for pediatric *H. pylori* infection, with P-CAB considerations. The pathway begins with confirmed infection and treatment indications. Initial regimen choice is guided by antibiotic susceptibility testing availability, leading to tailored or empirictherapy. Eradication success/failure is determined by^13^C-UBT or stool antigen test performed ≥4 weeks post-antibiotics and after acid-suppressant washout (per pediatric guidance). The bottom box lists key considerations for using P-CABs in first-line pediatrictherapy. P-CAB, potassium-competitive acid blocker; *H. pylori, Helicobacter pylori*; PPI, proton pump inhibitor; VPZ, vonoprazan; ^13^C-UBT, ^13^C-urea breath test. This pathway is intended to inform clinical reasoning rather than to supersede established local guidelines.

Antimicrobial susceptibility testing (AST) is necessary when conditions permit, but it typically requires invasive endoscopy, making widespread implementation difficult in pediatric practice. Consequently, the guidelines (Homan et al., 2024) endorse a 14-day bismuth-based quadruple therapy (bismuth, PPI, AMO, MET) as the empirical first-line eradication regimen when AST is not performed. Nevertheless, with the global increase in antibiotic resistance (Hirschl and Makristathis, 2007), the eradication rate of *H. pylori* with this approach remains suboptimal. In this context, P-CABs can be discussed as an alternative acid-suppressing backbone. The existing clinical evidence primarily centers on VPZ, which has demonstrated notable potential in first-line, second-line, and refractory *H. pylori* treatment. But pediatric evidence is still limited, relevant studies are summarized in **Table 1**. The relevant diagnostic and therapeutic flowchart is shown in **Figure 1**. **Figure 1** is intended as a pragmatic clinical decision pathway. It summarizes how treatment indication, AST availability, and regimen choice are integrated in pediatric clinical practice. The bottom section of the figure specifically outlines the scenarios where P-CAB-based regimens may be considered as a first-line option, highlighting key considerations such as regional clarithromycin resistance, adherence challenges, and the need for caution in younger children (aged <10 years) due to the paucity of direct evidence. This pathway is intended to inform clinical reasoning rather than to supersede established local guidelines.

**Table 1:** Key clinical evidence of VPZ–based regimens for pediatric *H. pylori* eradication.

Country and year	Study design	Population (n; age)	Indication for treatment	Method of diagnosis	Baseline resistance	Regimen (P-CAB arm)	Comparator	Test-of-cure and timing	Eradication rate
Japan 2018 (Kusano et al., 2018)	prospective observational study	n=118; median age 13.7 (13-16)	Junior high school students; *Hp*^+^ (GC prevention); Weight ≥ 40 kg; No allergy; Consent obtained	Initial: urine or stool or serum Ab^+^; Confirmation: ^13^C-UBT^+^	NR	VAC (VPZ 20 mg + AMO 750 mg + CLA 200 mg), BID, 7 days	None	^13^C-UBT at 8 weeks	81.3% (ITT)/85.7% (PP)
Japan 2019 (Kakiuchi et al., 2019)	Real-World Research	n=578; NR (≥ 15 years)	Age ≥ 15 years; *Hp*^+^ (GC prevention); consent obtained	Initial: urine Ab^+^; Confirmation: SAT (positive on both)	NR	First-line: VAC (VPZ 20 mg + AMO 750 mg + CLA 200 mg), BID, 7 days	None	^13^C-UBT at 8–12 weeks	85.1% (296/348)
Japan 2020 (Kaji et al., 2020)	Real-World Research	n=144; median age 14 (13-15)	Second-year junior-high school students; *Hp*^+^ (GC prevention); Consent obtained	Initial: urine Ab^+^; Confirmation: ^13^C-UBT^+^	NR	VAC (VPZ 20 mg + AMO 750 mg + CLA 200 mg), BID, 7 days	None	^13^C-UBT at 8 weeks	82.6% (ITT)/83.8% (PP)
Japan 2020 (Gotoda et al., 2020)	prospective observational study	n=221; NR (second-year junior-highschool students)	Second-year junior-highschool students; *Hp*^+^ (GC prevention); Consent obtained	Initial: urine Ab^+^; Confirmation: ^13^C-UBT^+^	NR	Dual therapy:VA (VPZ 20 mg + AMO 750 mg), BID, 7 days	Triple therapy:VAC (VPZ 20 mg + AMO 750 mg + CLA 200 mg), BID, 7 days	^13^C-UBT at 8 weeks	VA: 85.0% (ITT)/86.4% (PP) Vs VAC: 82.0% (ITT)/84.1% (PP)
Japan 2021 (Kakiuchi et al., 2021)	prospective observational study	n=390; NR (third-grade junior high school students)	Third-grade junior high school students; *Hp*^+^ (GC prevention); Weight ≥ 30^+^kg; No allergy; No liver/renal dysfunction; No mononucleosis; Consent obtained	Initial: urine Ab^+^; Confirmation: SAT (positive on both)	NR	First-line: VAC (VPZ 20 mg + AMO 750 mg + CLA 200 mg) + MIYA-BM, BID, 7 days	None	^13^C-UBT at 8 weeks	86.8% (ITT/PP)
Japan 2023 (Kakiuchi et al., 2023)	prospective observational study	n=645; mean age 16.9	Third-grade junior high school students; *Hp*^+^ (GC prevention); Weight ≥ 30^+^kg; No allergy; No liver/renal dysfunction; No mononucleosis; No colchicine use; Consent obtained	Initial: urine Ab^+^; Confirmation: SAT (positive on both)	NR	First-line: VAC (VPZ 20 mg + AMO 750 mg + CLA 200 mg), BID, 7 days	None	^13^C-UBT at 8–12 weeks	73.6% (ITT)/84.0% (PP);
USA 2025 (Cardenas Fernandez et al., 2025)	Single-Center Case Series	n=11; mean age 14.4 ± 3.4	Refractory *Hp*^+^ (≥ 1 prior treatment failure with good adherence)	Positive on both histopathology and culture	CLA: 50% (4/8); levofloxacin: 25% (2/8); MET: 75% (6/8)	VA (VPZ 20 mg BID + AMO 1000 mg TID), 14 days	None	82% SAT and 18% endoscopy/culture at 6–8 weeks	100% (11/11)
Egypt 2025 (Sa et al., 2025)	Randomized Controlled Trial	n=242; mean age 13.48 ± 2.52	Age 10–18 years; *Hp*^+^; Either: (a) dyspeptic symptoms (heartburn, dyspepsia, nausea, epigastric pain); (b) *Hp*^+^ gastritis; or (c) other indications per guidelines	Positive on two of: ^13^C-UBT, SAT, RUT, or histopathology	NR	VAC (VPZ 20 mg BID + AMO 50 mg/kg/day + CLA 20 mg/kg/day), 14 days	EAC (Esomeprazole [20 mg/day if < 30 kg and 40 mg/daily if ≥ 30 kg] + AMO 50 mg/kg/day + CLA 20 mg/kg/day), 14 days	^13^C-UBT or two-step monoclonal SAT at 4 weeks	VAC: 87.6% (ITT)/92.2% (PP) vs EAC: 76.9% (ITT)/83% (PP)

Potassium-Competitive Acid Blocker, P-CAB; VPZ, vonoprazan; AMO, amoxicillin; CLA, clarithromycin; MET, metronidazole; VA, VPZ + AMO dual therapy; VAC, VPZ + AMO + CLA triple therapy; *Hp*^+^, *Helicobacter pylori*-positive; GC, gastric cancer; Ab,antibody; ITT, intention-to-treat; PP, per-protocol; ^13^C-UBT, ^13^C-urea breath test; ^13^C-UBT^+^, ^13^C-urea breath test positive; EAC, Esomeprazole + AMO + CLA; BID, twice daily; TID, three times daily; SAT, stool antigen test; RUT, rapid urease test; MIYA-BM, probiotic; NR, not reported. Note, fixed doses were retained as reported in the source studies.

### 5.1 P-CAB-based triple therapy as first-line regimen

In recent years, empirical PPI-based triple therapy has shown suboptimal performance in pediatric practice, largely due to antibiotic resistance. Okuda et al., in an investigation of *H. pylori* eradication therapy in Japanese children, reported an eradication rate of 73.1% among 332 children receiving standard triple therapy as first-line treatment (Okuda et al., 2017). According to data from the European Society for Pediatric Gastroenterology, Hepatology and Nutrition (ESPGHAN) registry, the eradication rate of standard triple therapy (PPI + AMO + CLA) in children was only 61.8% (Kusano et al., 2018), which is far below the ideal level. The emergence of P-CABs has introduced new therapeutic strategies. VPZ-based triple therapy has become an important first-line treatment option for older pediatric patients (particularly adolescents) with *H. pylori* infection in regions such as Japan. Several large real-world studies have shown that its ITT eradication rate remains consistently between 81.3% and 85.1%. A randomized, double-blind controlled trial (the VONTAPE study) conducted in Egypt in 2025 confirmed that the eradication rate of VPZ-based triple therapy (ITT 87.6%) was significantly higher than that of esomeprazole-based triple therapy (ITT 76.9%) in individuals aged 10–18 years (Sa et al., 2025). The eradication rate of VPZ triple therapy is even comparable to that of susceptibility-guided bismuth quadruple therapy (Botija et al., 2024; Do et al., 2024). This advantage is particularly pronounced in regions with high CLA resistance (Isomoto et al., 2026). For example, a Japanese school-based screening project found that switching the first-line regimen from PPI-based triple therapy to VPZ-based triple therapy increased the eradication rate from 45.0% to 83.8% (Kaji et al., 2020), demonstrating the clinical value of P-CABs in overcoming CLA resistance. Moreover, the observed improvement in eradication rates with VPZ-based triple therapy does not imply that replacing the acid suppressant alone can fully overcome the limitations imposed by antibiotic resistance. A more conservative interpretation is that P-CABs may optimize the antibacterial environment and enhance the efficacy of co-administered antibiotics, but they cannot negate the fundamental challenge of resistance.

### 5.2 P-CAB-based dual therapy: reducing antibiotic exposure

In response to the global antibiotic resistance crisis, the importance of reducing unnecessary antibiotic exposure in pediatric patients has gained widespread recognition. Currently, *H. pylori* resistance to AMO remains significantly lower than that to CLA or MET (Li et al., 2017; Dutta et al., 2024; Hong et al., 2024; Menbari et al., 2025; Wu et al., 2025). Consequently, dual therapy using potent acid suppression plus high-dose AMO is an appealing antibiotic-sparing strategy. In adult patients, the clinical efficacy of PA (PPI + AMO) dual therapy has been controversial, with eradication rates ranging widely from 72.2% to 95.3% (Bayerdörffer et al., 1995; Yang et al., 2015; Kwack et al., 2016), and heavily influenced by dosage and treatment duration. Some studies reported suboptimal eradication rates (below 80%) (Graham et al., 2010; Kwack et al., 2016). In studies of adult patients, the eradication rate of VA (VPZ + AMO) dual therapy reached 87.1% (95% CI 81.0-91.8%), demonstrating non−inferiority to VAC (VPZ + AMO + CLA) triple therapy (Suzuki et al., 2020). This approach also avoids the pharmacodynamic antagonism between CLA and AMO and reduces antimicrobial resistance pressure (Drago et al., 2011), showing superior efficacy compared to PPI-based dual therapy. A Japanese prospective observational study demonstrated promising results in children. The 7-day VA dual therapy achieved a comparable eradication rate (ITT 85.0%) to the 7-day standard triple therapy (ITT 82.0%), meeting the non-inferiority criteria (ITT, p = 0.018; PP, p = 0.020) (Gotoda et al., 2020). This suggests that in populations with high CLA resistance, a CLA-free dual regimen could be considered for initial therapy, potentially ensuring efficacy while helping to curb resistance. For refractory infections after multiple treatment failures, a U.S. case series reported an even more aggressive strategy. Utilizing VPZ combined with ultra-high-dose, high-frequency AMO (1000mg, three times daily) for 14 days achieved a 100% eradication rate in 11 children who had failed previous multiple therapies (Cardenas Fernandez et al., 2025). This provides a new avenue for managing the most challenging cases. VA dual therapy appears to be a promising alternative to triple therapy in selected older pediatric cohorts with *H. pylori* infection. It represents a novel strategy capable of achieving adequate eradication rates while reducing antibiotic use, which is crucial for preventing future antimicrobial resistance in *H. pylori*. However, key uncertainties remain, including the optimal dose and dosing frequency of AMO, as well as the ideal treatment duration for this regimen in pediatric patients.

### 5.3 P-CABs in salvage therapy

For treatment failure, AST should be pursued whenever feasible to guide salvage therapy.

Available pediatric data suggest that VPZ-based salvage regimens can achieve high eradication rates when antibiotics are appropriately switched after first-line failure. Regarding efficacy, for cases failing first-line therapy, P-CAB-based salvage regimens achieve high eradication rates of 92.8% to 100% (Gotoda et al., 2020; Kakiuchi et al., 2021; Kakiuchi et al., 2023). They demonstrate particular superiority against CLA-resistant, multidrug-resistant strains, or refractory infections (≥ 1 prior treatment failure). A US single-center study, though small in scale, reported a 100% eradication rate in children with refractory infections (who had failed prior PPI triple or bismuth quadruple therapy) using VA (14 days) (Cardenas Fernandez et al., 2025), providing encouraging preliminary evidence. Japanese multicenter data confirmed that after failure of a first-line P-CAB and CLA regimen, switching to a second-line P-CAB and MET regimen achieved eradication rates of 92.8% to 97.3%. Even the rare cases failing second-line therapy could be completely eradicated using a third-line P-CAB and sitafloxacin regimen (Kakiuchi et al., 2019). In a specific instance, 15 children who failed initial eradication with PAC triple therapy were treated with PAM (P-CAB + AMO + MET) salvage triple therapy, achieving a 100% eradication rate (Kusano et al., 2018), suggesting that the initial failure might have been related to CLA resistance. From a treatment strategy perspective, the P-CAB component itself often does not need to be changed following first-line failure. Merely adjusting the accompanying antibiotics (e.g., replacing CLA with MET) can achieve satisfactory efficacy.

### 5.4 Treatment adherence in P-CAB-based regimens for *H. pylori* eradication

Adherence is a major determinant of eradication success in pediatrics. Beyond choosing an effective regimen, clinicians should address practical barriers (K et al., 2017; Le Thi et al., 2023). In pediatric populations, missed doses, premature discontinuation, or incorrect dosing (timing or method) significantly compromise eradication rates. PPI-based regimens impose stricter administration requirements compared to P-CABs. A clinical study in adolescents identified both treatment adherence and the use of VPZ (vs. PPI, p = 0.019, OR = 2.34, 95% CI: 1.15–4.91) as the only significant independent factors influencing therapeutic success (Sa et al., 2025). This underscores the advantageous role of P-CABs within combination regimens. Available study data indicate that medication completion rates for P-CAB-based combination therapies in children generally exceed 90%. Nonetheless, pill burden and antibiotic intolerance remain key drivers of non-completion. Beyond efficacy, the intrinsic usability advantages of P-CABs—including smaller tablet size for easier swallowing and administration flexibility regardless of food intake—provide crucial, practical support for enhancing treatment adherence in the clinical management of pediatric patients.

### 5.5 Cost - effectiveness considerations

To date, no pediatric cost-effectiveness study comparing P-CAB- and PPI-based eradication regimens has been published. In adult populations, evidence from Japan indicates that VPZ-based triple therapy is more cost-effective than PPI-based triple therapy (Kajihara et al., 2017; Seko et al., 2019). Lu et al. also showed that, in Chinese patients, a VPZ-containing quadruple therapy had a much lower cost-effectiveness ratio (1.32-1.88) than an esomeprazole-based quadruple therapy (3.06) (Lu et al., 2023). However, one US study concluded that bismuth quadruple therapy was more cost-effective (Liu et al., 2026). Although these adult findings are informative, significant differences between children and adults in drug metabolism, weight-based dosing, and reimbursement policies preclude direct extrapolation. However, P-CABs may still reduce treatment costs by shortening treatment duration and simplifying regimens. Future pediatric studies should specifically evaluate the cost-effectiveness of P-CABs for *H. pylori* eradication, taking into account local resistance patterns and reimbursement policies.

## 6 Safety and tolerability of P-CABs in *H. pylori* eradication therapy for children

In pediatric eradication studies, VPZ exposure is short (typically 7–14 days) and safety reporting has focused on short-term adverse events (AEs) during therapy and shortly after completion. Across published VPZ pediatric studies, short-term tolerability appears similar to PPI-based regimens, with few serious AEs reported.

Reported incidences of AEs vary considerably across studies, ranging from 4.0% to 36.9% (**Table 2**). This variation is likely attributable to differences in the rigor of monitoring methods. Studies employing patient-completed daily symptom diaries tend to capture transient symptoms more sensitively and report significantly higher AE rates compared to studies relying solely on recall or questioning during clinic visits (Kakiuchi et al., 2021; Kakiuchi et al., 2023).

**Table 2:** AEs reported in pediatric studies of VPZ-based *H. pylori* eradication.

Country and year	Regimen (P-CAB arm)	N	Overall AEs (%)	Common AEs (n/%)	Serious AEs (n/reason)	Discontinuation due to AEs (n/reason)
Japan 2018 (Kusano et al., 2018)	VAC (VPZ 20 mg + AMO 750 mg + CLA 200 mg), BID, 7 days	118	21.10%	Loose stools (8/7.2%), diarrhea (7/6.3%), rash (5/4.5%), vomiting (2/1.8%), abdominal pain (2/1.8%), and dysgeusia (1/0.9%).	7 (rash in five, vomiting in two)	7 (drug allergy)
Japan 2019 (Kakiuchi et al., 2019)	VAC (VPZ 20 mg + AMO 750 mg + CLA 200 mg), BID, 7 days	501	4.00%	Diarrhea (7/1.4%), abdominal pain (6/1.2%), nausea/vomiting (6/1.2%), urticaria (3/0.6%), fever (2/0.4%), and dysgeusia (1/0.2%).	NR	4 (drug intolerance)
Japan 2020 (Kaji et al., 2020)	VAC (VPZ 20 mg + AMO 750 mg + CLA 200 mg), BID, 7 days	142	9.80%	Diarrhea (10/7.0%), abdominal pain (2/1.4%), abdominal distension (1/0.7%), and nausea (1/0.7%).	NR	NR
Japan 2020 (Gotoda et al., 2020)	VA (VPZ 20 mg + AMO 750 mg), BID, 7 days	60	10.00%	Diarrhea (2/3.3%), abdominal pain (2/3.3%), and rash (1/1.7%).	NR	NR
	VAC (VPZ 20 mg + AMO 750 mg + CLA 200 mg), BID, 7 days	161	19.80%	Loose stools (11/6.8%), diarrhea (7/4.5%), abdominal pain (1/0.6%), nausea/vomiting (3/1.8%), and rash (7/4.3%).	NR	2 (rash and abdominal pain)
Japan 2021 (Kakiuchi et al., 2021)	VAC (VPZ 20 mg + AMO 750 mg + CLA 200 mg)+ MIYA-BM, BID, 7 days	274	NR	Diarrhea (117/47.2%), abdominal pain (78/28.5%), nausea and vomiting (3/1.1%), rash (drug eruption) (3/1.1%), stomatitis (1/0.4%), headache and malaise (1/0.4%)	NR	2 (diarrhea, 8 defecations per day, and urticaria)
Japan 2023 (Kakiuchi et al., 2023)	VAC (VPZ 20 mg + AMO 750 mg + CLA 200 mg) + MIYA-BM, BID, 7 days	588	NR	Abdominal pain (164/27.9%), diarrhea (217/36.9%), nausea or vomiting (7/1.2%), and urticaria (13/2.2%).	NR	5 (diarrhea in 3 cases, abdominal pain in 1 case, urticaria in 1 case)
	VAM (VPZ 20 mg + AMO 750 mg + MET 200 mg), BID, 7 days	62	NR	Abdominal pain (12/19.4%) and diarrhea (17/27.4%).	NR	NR
USA 2025 (Cardenas Fernandez et al., 2025)	VA (VPZ 20 mg BID + AMO 1000 mg TID),14 days	11	18.00%	Nausea (1/9%) and diarrhea (1/9%).	NR	NR
Egypt 2025 (Sa et al., 2025)	VAC (VPZ 20 mg BID + AMO 50 mg/kg/day + CLA 20 mg/kg/day), 14 days	121	16.50%	Diarrhea (7/5.8%), nausea (8/6.6%), and abdominal colic (4/3.3%).	NR	NR

The most commonly reported adverse events are gastrointestinal (e.g., diarrhea/loose stools, abdominal discomfort), consistent with the profile of co-administered PPI (Saito et al., 2025), which are largely attributable to the antibiotic backbone rather than the acid suppressant. Nausea/vomiting and rash occur relatively less often (typically <10%), with rashes frequently related to beta-lactam allergy (Kusano et al., 2018; Gotoda et al., 2020). Head-to-head pediatric data suggest no meaningful difference in overall AE frequency between VPZ- and PPI-based triple therapy (Sa et al., 2025). Most symptoms are self-limited and resolve after therapy completion.

Previous studies suggest that probiotics are effective in reducing abdominal AEs related to *H. pylori* eradication (Çekin et al., 2017; Handa et al., 2020). A meta-analysis of randomized controlled trials concluded that *Lactobacillus* improved *H. pylori* eradication success while reducing the incidence of eradication-related diarrhea in children (Fang et al., 2019), a finding consistent with results in adults (Hauser et al., 2015). However, in studies involving VPZ combined with antibiotics for *H. pylori* eradication (Kakiuchi et al., 2021; Kakiuchi et al., 2023), the addition of probiotics was not observed to significantly ameliorate gastrointestinal reactions. This may be related to their evaluation methods and a lack of control groups without probiotics, thus limiting their reference value. Nonetheless, in clinical practice, adding probiotics or gastric mucosal protective agents to eradication regimens may still be considered to potentially enhance patient tolerability and eradication rate (Šterbenc et al., 2026), as the potential benefits may outweigh the risks.

*H. pylori* infection and eradication therapy can perturb the gut microbiome in children, and antibiotics are the dominant driver of short-term dysbiosis (Ye et al., 2020; Zheng et al., 2021; Zhou et al., 2021). Although PPI-based eradication regimens may exacerbate microbiota disturbances in the short term, most microbiota indicators can return to baseline levels approximately one year after the completion of treatment (Zhou et al., 2021). Given the similar mechanism of action, P-CABs theoretically carry comparable risks. However, adult studies have shown that the α-diversity or microbial composition of the gut microbiota in the P-CAB-based VA dual therapy group showed no significant difference from baseline values one month post-treatment. In contrast, the PPI group still exhibited incomplete restoration of microbiota structure one month after treatment (Hu et al., 2023). Horii et al. reported that compared to VAC triple therapy, VA dual therapy induced minimal alterations in both the diversity and relative abundance of gut microbiota (Horii et al., 2021), which suggests that reducing antibiotic use may have a protective effect on the gut microbiota. However, there are currently no reported studies on the effects of P-CABs on gut microbiota in the pediatric population. Moving forward, pediatric studies ought to incorporate changes in microbiome structure—particularly as pre-specified endpoint measures.

Currently, all published clinical studies on *H. pylori* treatment in children focus exclusively on VPZ. There is a lack of efficacy, comparative data, or sufficient safety evidence from clinical studies for tegoprazan or other P-CABs in the pediatric population. Therefore, the efficacy and safety data for VPZ currently serve as the sole evidence guiding clinical practice for children. The potential application of other P-CABs in pediatrics awaits future exploration. However, no AEs unique to children have been observed to date, and there have been no reported cases leading to serious conditions such as death or sequelae (Kusano et al., 2017; Kakiuchi et al., 2019; Kaji et al., 2020).

In summary, VPZ-based treatment regimens exhibit a favorable safety and tolerability profile in pediatric patients. Nevertheless, it must be emphasized that the available evidence is largely confined to older children and adolescents; consequently, these safety conclusions should not be uncritically extrapolated to younger children. The observed adverse events are primarily driven by the antibiotic backbone of the regimen, and serious adverse events are uncommon. But inter-study AE rates are not directly comparable because of heterogeneity in ascertainment methods, antibiotic backbones, treatment duration, and study design.

## 7 Challenges and future perspectives

Research on the application of P-CABs in the treatment of *H. pylori* infection in children has made preliminary progress. Although most studies have still not achieved the target eradication rate of 90%, there has been significant improvement compared to traditional PPI-based combination regimens (Al Atrash et al., 2025). However, significant evidence gaps and key unresolved issues remain in this field. Firstly, published studies have predominantly focused on adolescent or near-adult populations (typically aged ≥10 years), whereas clinical data on the use of P-CABs for anti-*H. pylori* therapy in preschool and school-aged children (aged <10 years) are almost entirely absent. This concentration of evidence in a narrow age range limits the feasibility of meaningful age-stratified analyses and precludes assessment of potential age-related differences in treatment response, safety, and tolerability. Given the physiological and developmental distinctions between younger children and adolescents, this evidence gap underscores the need for future studies that encompass a broader pediatric age spectrum to allow more robust evaluation across different developmental stages. Secondly, pediatric dosing of P-CABs has largely relied on directly adopting fixed adult dosing schemes (e.g., for VPZ, 20 mg twice daily for patients ≥12 years and weighing >30 kg). This empirical approach lacks support from targeted pharmacokinetic studies, carrying a high risk of overdosing or underdosing in children with lower body weight. Future research should focus on conducting pharmacokinetic-pharmacodynamic studies in children stratified by age and weight to establish the quantitative relationship between plasma drug concentration and acid-suppressive effect, thereby facilitating the development of scientifically sound and standardized individualized dosing regimens. Regarding the scope of drug applicability, the current pediatric clinical evidence is limited exclusively to VPZ. Other P-CABs already used in adult clinical practice (e.g., keverprazan, tegoprazan) have not been studied for *H. pylori* eradication in children, highlighting an urgent need to expand the spectrum of drugs investigated in this field. Furthermore, the existing available evidence primarily originates from Japanese populations. Data on the use of P-CABs for eradication therapy in children from Western countries have not been publicly reported. Considering the marked differences in *H. pylori* epidemiology, antibiotic resistance patterns, and population genetic/metabolic backgrounds across regions, there is a pressing need for international, multi-center collaborative studies. Such efforts are essential to validate the efficacy and safety of P-CAB-based treatment regimens in children of diverse ethnicities across Europe, the Americas, Africa, and other regions, thereby filling geographical data gaps and enhancing the global representativeness of research conclusions. Regrettably, AST data remain scarce in the pediatric literature. This limitation is largely attributable to the invasive nature of AST, which requires gastric mucosal biopsy obtained via endoscopy. Consequently, the current evidence base lacks systematically collected AST data, limiting the ability to precisely characterize resistance patterns and to correlate them with eradication outcomes in pediatric populations. Moreover, compared with traditional PPIs, P-CABs have higher drug costs, which may limit their use in some regions. Existing clinical evidence does not report the cost-effectiveness of eradication regimens in pediatrics, and future efforts should strive to fill this gap. Notably, there is a lack of systematic follow-up data on the long-term effects in children receiving P-CAB therapy, such as changes in gastric mucosal morphology, fluctuations in serum gastrin levels, and *H. pylori* recurrence rates. An ideal research model would involve establishing long-term follow-up cohorts of children post-*H. pylori* eradication therapy. Through several years of longitudinal observation, trends in gastritis recrudescence risk, reinfection rates, and changes in growth and development indicators could be clarified, providing evidence-based support for the long-term use of P-CABs in the pediatric population.

## 8 Conclusion

P-CABs, particularly VPZ, may offer practical advantages in selected older pediatric populations by simplifying administration and improving acid suppression. However, the current evidence base remains limited, heterogeneous, and heavily weighted toward adolescents, and broader recommendations should await more robust pediatric data.
